# Urothelial genotoxicity of household chemicals in healthy canine urinary bladder organoids relative to observed urinary exposures in pet dogs

**DOI:** 10.3389/fvets.2025.1702980

**Published:** 2025-12-23

**Authors:** Hannah M. Peterson, Christopher Zdyrski, Karin Allenspach, Jonathan P. Mochel, Lauren A. Trepanier

**Affiliations:** 1 Department of Medical Sciences, University of Wisconsin-Madison, Madison, WI, United States; 2 Department of Pathology, University of Georgia, Athens, Georgia; 3 Precision One Health Initiative, University of Georgia, Athens, Georgia; 4 Office of Biotechnology, Iowa State University, Ames, IA, United States

**Keywords:** comet assay, DNA damage, bladder cancer, aromatic amines, arsenic, acrolein

## Abstract

**Introduction:**

Urothelial carcinoma (UC) in pet dogs closely resembles human muscle-invasive UC, which is associated with environmental chemical carcinogens. The aim of this study was to determine whether urinary concentrations of the bladder carcinogens acrolein, inorganic arsenic, and 2,6-dimethylaniline (2,6-DMA) reach genotoxic concentrations in pet dogs with and without UC.

**Methods:**

We first established thresholds for DNA damage from these chemicals using a novel *in vitro* organoid model. Healthy canine urinary bladder organoids were exposed to acrolein, sodium arsenite, and 2,6-DMA *in vitro* and we used the alkaline CometChip assay without and with the enzyme Fpg (*formamidopyrimidine [fapy]-DNA glycosylase*) to measure DNA strand breaks and oxidative DNA damage.

**Results:**

For acrolein, we found a genotoxic threshold of 20 uM for combined DNA strand breaks and oxidative DNA damage. These findings suggest potentially genotoxic urinary acrolein exposures in 20% of pet dogs (15 of 74) previously surveyed, with no differences between cases and controls. For inorganic arsenic, we observed genotoxicity at 20 uM in canine organoids; none of 74 pet dogs reached this urinary concentration when assayed at a single time point. For 2,6-DMA, the genotoxic threshold was 0.01 uM for combined DNA strand breaks and oxidative DNA damage. Among dogs previously surveyed, 8% of UC cases (3 of 37) and none of 36 controls reached this threshold (*p* = 0.07).

**Conclusion:**

Acrolein and 2,6-DMA could reach genotoxic urinary concentrations after household exposures in some pet dogs, and the role of 2,6-DMA in canine bladder cancer risk deserves assessment in a larger sample size.

## Introduction

Urothelial carcinoma (UC) is the most common type of bladder cancer in pet dogs, affecting an estimated 20,000 dogs per year (1). Canine UC can lead to hematuria, dysuria, and urinary obstruction, and it typically results in euthanasia because of deteriorating quality of life. While certain breeds are at higher risk for UC (2), environmental factors are also important (3–5). Notably, muscle-invasive UC in people resembles canine UC in clinical behavior, histopathology, molecular characteristics, and poor prognosis (6). Human UC has been associated with exposures to acrolein, inorganic arsenic, and aromatic amines (7–9).

Healthy pet dogs have urinary exposures to acrolein and inorganic arsenic that are 4- to 6-fold higher than those of their owners (10), and the aromatic amine 2,6-dimethylaniline (2,6-DMA) was identified as the most prevalent aromatic amine in dog urine (11). Dogs with UC also exhibit urinary exposures to these chemicals (12, 13), but it remains unclear whether these exposures reach genotoxic concentrations. Genotoxicity from inorganic arsenic and acrolein has previously been characterized in immortalized canine cell lines and in a single primary canine urothelial cell line (14). However, oxidized DNA damage was not assessed, and 2,6-DMA was not evaluated. Furthermore, canine bladder organoids might provide a better *in vitro* model for urothelial genotoxicity because they recapitulate the cellular heterogeneity and biologic behavior of native urothelial cells (15).

The purpose of this study was to characterize the genotoxicity of acrolein, inorganic arsenic, and 2,6-DMA in a novel *in vitro* canine urinary organoid model (16), assessing both DNA strand breaks and oxidized DNA residues, and to determine whether urinary chemicals reached genotoxic concentrations in pet dogs from two recent case–control studies (12, 13).

## Methods

### Canine urinary bladder organoids

Three canine organoid lines were used that were derived from healthy bladder tissue obtained from an intact female beagle at Purdue University and cultured at the University of Georgia College of Veterinary Medicine, in addition to two intact female beagle-mix dogs collected and cultured at Iowa State University, following approved IACUC protocols. The organoid lines were cultured and maintained at the University of Georgia using established protocols (17); subsets of these bladder organoids have been previously characterized both by morphology and immunohistochemistry, bulk RNASeq, and scRNASeq to establish expression of uroplakins, indicative of differentiated urothelial umbrella cells (17). Urinary bladder organoids were shipped frozen on dry ice to the University of Wisconsin-Madison and were stored in liquid nitrogen (−196 °C) for up to 2 weeks before thawing and expansion for use.

Thawed organoids were resuspended for culture in a 24-well plate in Matrigel® (Corning® Life Sciences, cat. #356231), a solubilized murine basement membrane preparation that contains growth factors and extracellular matrix proteins (18). Organoids were maintained at 37 °C in CMGF+ (complete medium with growth factors) containing inhibitors of ROCK (Rho-associated protein kinases; 10 μM; Y-27632, Biogems, cat. # 1293823) and GSK3β (glycogen synthase kinase 3 beta; 2.5 μM; Stemolecule CHIR99021, Stemgent; cat. # 04–0004) (17). After thawing, organoids were expanded through two additional passages to final passage numbers of 4 (Line 1) and 8 (Lines 2 and 3). To produce a single-cell suspension for use in *in vitro* DNA damage assessments, the organoids were dissociated using TrypLE™ Express (ThermoFisher; cat. #12604013) for 10 min and passed through 40-micron filters to remove clumps. Individual live cells were counted with a hemocytometer using Trypan blue dye exclusion and were immediately used for *in vitro* DNA damage assays.

### DNA damage assessment

To assess DNA damage *in vitro*, we used a high-throughput adaptation of the traditional alkaline comet assay, the CometChip (19), which detects single- and double-stranded DNA breaks (20). The CometChip platform utilizes 96-well agarose gel plates embedded with microwells to create a single-cell microarray. This format minimizes cell clumping, improves cell distribution, and minimizes inter- and intra-assay variability (19). We initially fabricated CometChips using a 30-micron stamp generously provided by Dr. Bevin Engelward, using a protocol developed by her team at the Massachusetts Institute of Technology (21). In later stages of the study, pre-made CometChips were supplied by Dr. Engelward’s laboratory (CellArray, Lexington, MA).

For chemical exposure, cells (~100,000 per well) were incubated for 30 min at 37 °C with 5% CO_2_ to allow settling into microwells. Cells were washed with 1X PBS, overlaid with 1% low-melting point agarose in 1X TBE buffer, and allowed to solidify. Each chemical—acrolein (0 to 56 uM; Restek, Bellafonta, PA; cat. #30646), sodium arsenite (0 to 75 uM; Sigma-Aldrich, St. Louis, MO; cat #S9663), or 2,6-DMA (0.01 to 1,000 uM, Sigma-Aldrich, St. Louis, MO; cat. # 442327)—was added to wells and incubated for 6 h (14) for each canine organoid line. After chemical exposures, chips were immersed in an alkaline lysis solution (2.5 M NaCl, 100 mM Na_2_EDTA, 10 mM Trizma® Base, and 1% Triton X-100, pH 10) at 4 °C overnight.

Prior to electrophoresis, chips were submerged three times for 5 min each in enzyme buffer (40 mM HEPES, 0.1 M KCl, 0.5 mM EDTA, 0.2 mg/mL BSA, pH 8). Half of the wells had Fpg enzyme (1 ug/mL final concentration; St. Louis, MO, Sigma-Aldrich; cat. #F3174) added to buffer during a 30-min incubation at 37 °C. Fpg (*formamidopyrimidine [fapy]-DNA glycosylase*) detects oxidized DNA base modifications and converts them into DNA single-stranded breaks that can be detected by the comet assay (22). The remaining wells were incubated with enzyme buffer alone for 30 min at 37 °C, to measure direct chemically induced DNA strand breaks. After incubation, wells were washed with 1X PBS and exposed to alkaline unwinding buffer (0.3 M NaOH and 1 mM Na_2_EDTA) for 40 min at 4 °C. The DNA was electrophoresed in the same buffer for 30 min at 1 V/cm and ~300 mA. Chips were submerged twice in neutralization buffer (0.4 M Trizma® base at pH 7.5) for 15 min each, washed with deionized water and then 70% ethanol, and dried overnight before imaging.

**Figure 1:**
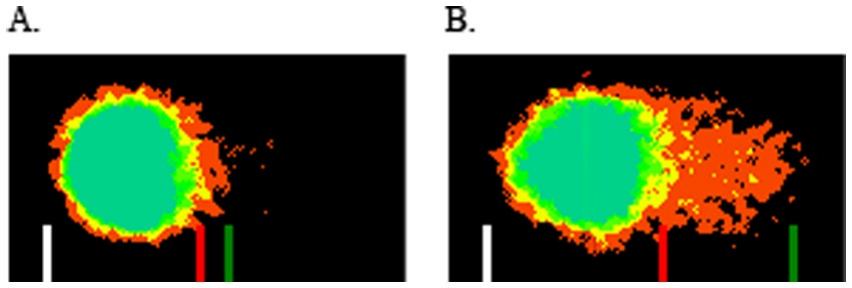
Nuclear DNA from canine urinary bladder organoids exposed to acrolein and assessed for DNA damage using the alkaline CometChip assay. Electrophoresed DNA was stained with SYBR™ Gold, and DNA damage was calculated as % DNA in the “comet tail” (between red and green lines) using CometAssay Analysis Software. **(A)** Canine urinary organoid line 3 exposed to vehicle for 6 h. **(B)** Canine urinary organoid line 3 exposed to 56 uM acrolein for 6 h.

Cellular DNA in the CometChip wells was stained for 30 min at room temperature with 2X SYBR™ Gold in 1X TBE buffer. Imaging was performed with the Leica TCS SP8 laser scanning confocal microscope with a 4X objective with a 495-nm excitation filter. DNA strand breaks (as percent DNA in the comet tails) were quantified using CometAssay Analysis Software (Bio-Techne, Minneapolis, MN), with at least 50 cells counted per replicate. Each experiment was performed in triplicate on two separate occasions. Data were expressed as mean percent DNA in the comet tail (Figure 1) (20). Oxidized DNA was calculated by subtracting percent comet tail DNA in wells without Fpg (which reflects direct chemically-induced DNA breaks), from percent comet tail DNA in wells with Fpg (which reflects oxidized DNA plus chemically induced DNA strand breaks).

### Canine UC cases and controls

Pet dogs with UC and age-, breed-, and sex-matched unaffected controls were previously recruited nationally for a prospective case–control study of urinary chemical exposures (12). Cases included 37 dogs of various breeds, aged 8–16 years, with 21 spayed females, 13 neutered males, and 3 intact males. Unaffected controls included 37 dogs of various breeds, aged 8–18 years, comprising 22 spayed females, 1 intact female, 11 neutered males, and 3 intact males (12). Voided urine was assayed for inorganic arsenic species using coupled high-performance liquid chromatography and magnetic-sector inductively coupled plasma mass spectrometry (12). Because acrolein is unstable, its stable metabolite, 3-HPMA (*S*-3-hydroxypropyl mercapturic acid), was measured in urine, using liquid chromatography with tandem mass spectrometry (LC–MS/MS) (12). Banked urine samples from these dogs (minus one control dog that had insufficient remnant urine volume) were subsequently screened for aromatic amines, including 2,6-dimethylaniline, using LC–MS/MS (13).

### Statistical analyses

*In vitro* DNA damage, expressed as mean percent tail DNA in the CometChip assay, was compared between vehicle controls and increasing concentrations of each chemical using one-way ANOVA followed by Dunnett’s multiple comparison tests. This analysis established thresholds for DNA damage for acrolein, inorganic arsenic, and 2,6-dimethylaninine (DMA) in canine urinary bladder organoids, including DNA strand breaks, oxidative DNA base modifications, and combined DNA damage (both strand breaks and oxidized residues).

The proportions of pet dogs reaching genotoxic urinary levels for each chemical were determined from urinary chemical concentrations in two recent case–control studies (12, 13). These proportions of dogs reaching genotoxic urinary concentrations were then compared between UC cases and controls using Fisher’s exact tests. All statistical analyses were conducted using commercial software (Prism 10, GraphPad Software LLC), with *p* < 0.05 considered significant.

## Results

### Acrolein genotoxicity

**Figure 2:**
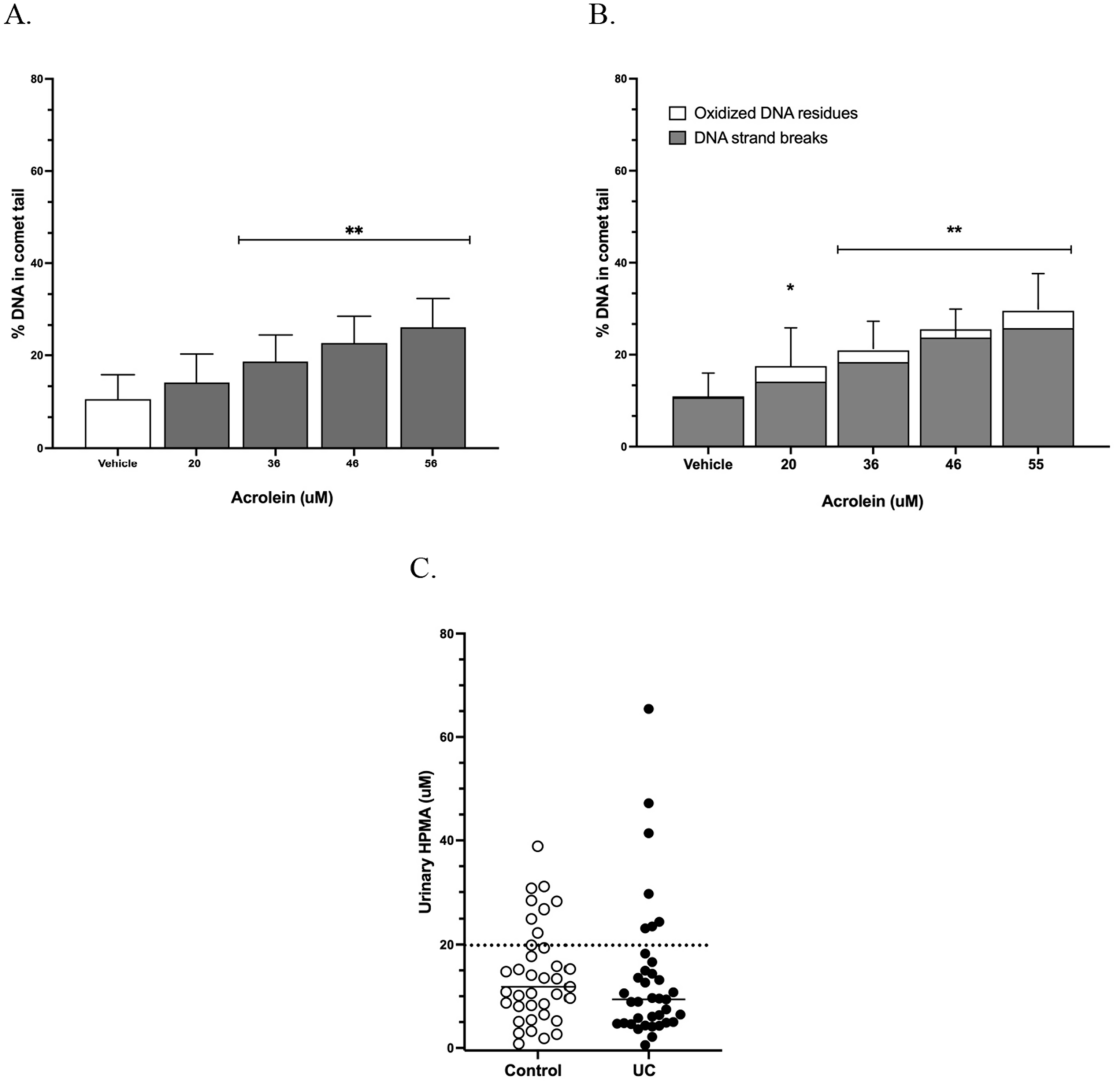
Urothelial genotoxic thresholds for acrolein in dogs. **(A)** Genotoxicity of acrolein in canine urinary organoids, measured as DNA strand breaks using the alkaline CometChip assay. **p ≤ 0.001 compared to vehicle. **(B)** Genotoxicity of acrolein as direct DNA strand breaks plus oxidative DNA base modifications using the CometChip assay with the addition of the enzyme Fpg. *p = 0.018; **p ≤ 0.0002. Data shown are from three individual canine urinary organoids, each assayed in triplicate on two occasions. **(C)** Urinary concentrations of the stable urinary acrolein metabolite, 3-HPMA (3-hydroxypropylmercapturic acid) previously measured in the urine of pet dogs with and without urothelial carcinoma (UC) (12). The dotted line indicates a 20 uM threshold for combined DNA damage from acrolein *in vitro*, with no significant difference between the number of cases and controls reaching this threshold (*p* ≥ 0.99).

Acrolein induced direct DNA strand breaks in canine urothelial cells at mean concentrations of ≥ 36 uM (Figure 2A). Additional oxidative DNA damage was detected during the 6-h incubation, which lowered the combined DNA damage threshold to 20 uM (Figure 2B). In pet dogs, urinary acrolein, measured as its stable metabolite 3-hydroxypropylmercapturic acid (3-HPMA), ranged from 0.6 to 65.4 uM (125–14,480 ng/mL) in UC cases and 0.8 to 38.9 uM (176–8,600 ng/mL) in unaffected controls (12). Assuming urinary 3-HPMA reflects urothelial acrolein exposure, the proportion of these pet dogs reaching a urinary genotoxic threshold of 20 uM was 18.9% for UC cases (7 of 37) and 21.6% for controls (8 of 37; *p* > 0.99; Figure 2C) (12).

### Arsenic genotoxicity

**Figure 3:**
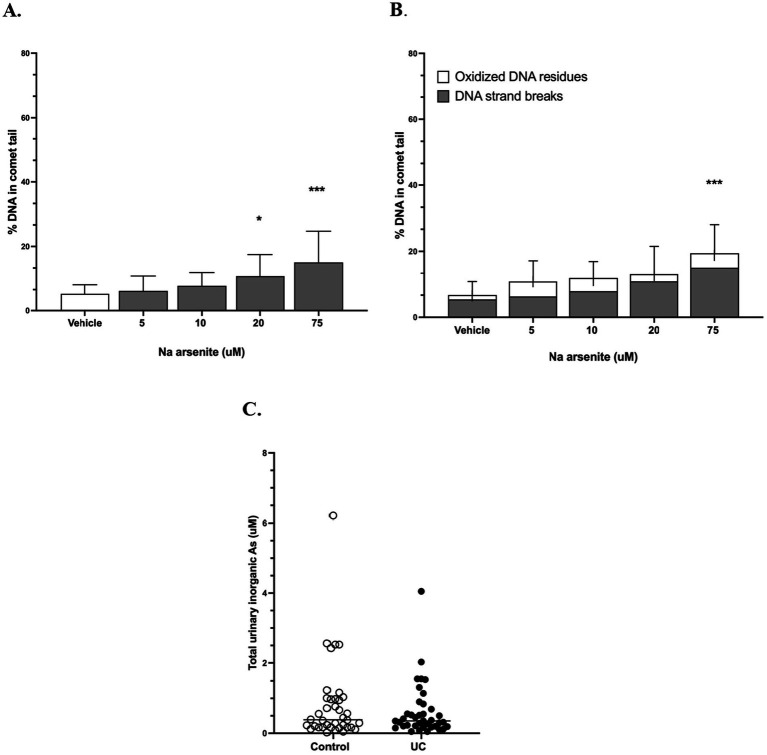
Genotoxicity of inorganic arsenic in canine urinary organoids. **(A)** Genotoxicity of sodium arsenite measured as direct DNA strand breaks using the alkaline CometChip assay. *p = 0.038; ***p < 0.0001. **(B)** Genotoxicity of sodium arsenite as direct DNA strand breaks plus oxidative DNA base modifications using the CometChip assay with the addition of the enzyme Fpg. ***p < 0.0001. Data shown are from three separate urinary organoids, each assayed on two occasions in triplicate. **(C)** Total urinary concentrations of inorganic arsenic species previously measured in pet dogs without and without urothelial carcinoma (UC) (12). No cases or controls reached 20 uM of total inorganic arsenic.

DNA strand breaks were observed with sodium arsenite at ≥ 20 uM (Figure 3A), while additional oxidative DNA damage was variable (Figure 3B). Total urinary inorganic arsenic concentrations ≥ 20 uM were not reached in any of 74 pet dogs in a recent canine UC case–control study (Figure 3C) (12).

### 2,6-dimethylaniline (2,6-DMA) genotoxicity

**Figure 4:**
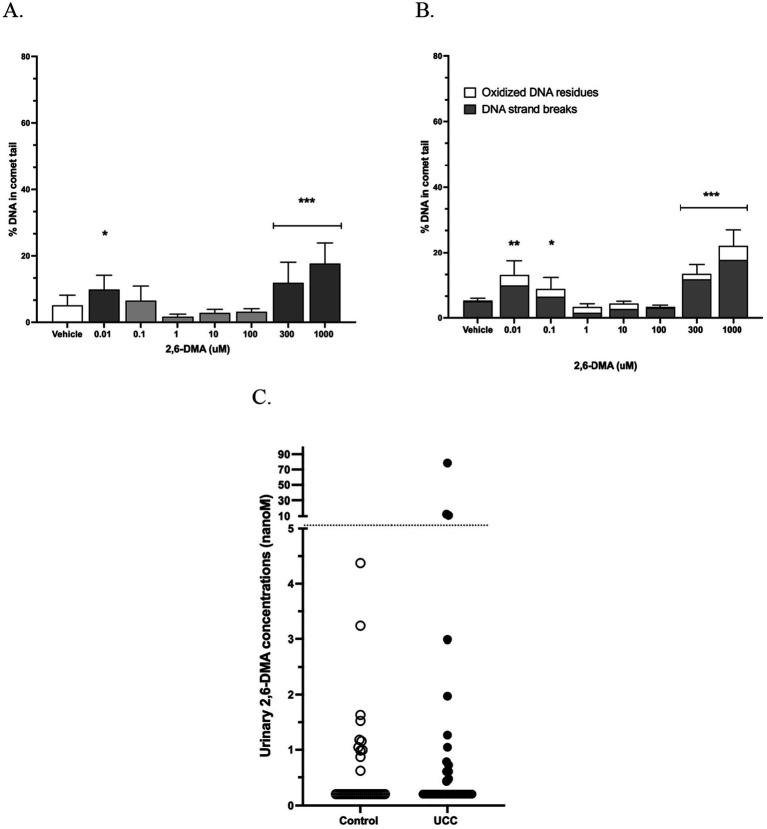
Urothelial genotoxicity of the aromatic amine 2,6-dimethylaniline (2,6-DMA) in dogs. **(A)** DNA strand breaks from 2,6-DMA in canine urinary organoids, measured using the alkaline CometChip assay. Data shown are from three separate urinary organoids, each assayed on two occasions in triplicate (concentrations between 0.1 and 100 uM could only be obtained in one set of organoids). *p = 0.04, ***p ≤ 0.0002. **(B)** DNA strand breaks plus oxidative DNA base modifications from 2,6-DMA in canine urinary organoids, measured using the CometChip assay with the addition of the enzyme Fpg. *p = 0.03, **p = 0.0008, ***p < 0.0001. **(C)** Urinary 2,6-DMA previously measured in the urine of pet dogs without and without urothelial carcinoma (UC) (13). The dotted line indicates 0.01 uM; 3 of 37 UC cases and none of 37 unaffected controls reached this urinary 2,6-DMA concentration.

The aromatic amine 2,6-DMA led to DNA strand breaks at concentrations of 0.01 uM (Figure 4A), although we did not observe concentration dependence through 100 uM. This is likely because experiments at 1 to 100 uM 2,6-DMA could only be obtained in one set of canine organoids. There was little additional oxidative DNA damage observed, which did not change the observed DNA damage thresholds (Figure 4B). In a recent case–control study of urinary aromatic amines in pet dogs (13), urinary 2,6-DMA concentrations ranged from 0.03 to 9.53 ng/mL (0.21–78.6 nM). Of these, 3 of 37 UC cases (8%) and none of 36 unaffected controls reached 2,6-DMA concentrations of 0.01 uM (10 nM) in their urine (*p* = 0.07; Figure 4C) (13).

## Discussion

Acrolein, inorganic arsenic, and 2,6-DMA all led to DNA strand breaks in canine urothelial cells, as assessed in healthy canine urinary bladder organoids. For acrolein, the mean genotoxic threshold in canine organoids was 36 uM for DNA strand breaks and 20 uM when oxidative DNA damage was included. These thresholds are consistent with those observed for direct strand breaks in immortalized canine urothelial and primary urothelial cell lines (36–56 uM) using the same CometChip assay (14). Although DNA strand breaks can result from cellular toxicity *in vitro* rather than direct interactions with DNA, tobacco condensate fractions that contain acrolein produce minimal cytotoxicity in human urothelial cells at concentrations up to 2.6 mM (23).

Human urothelial cells, including HT-1376, T-24, and primary cells, exhibit genotoxic responses to acrolein at substantially lower concentrations (1–3 μM) than canine cells under identical experimental conditions (14). Previous studies reported mutagenic effects at similarly low concentrations (2.5 uM) in human primary and immortalized urothelial cells (9). The higher genotoxicity thresholds observed in canine urothelial cells suggest species-related differences in sensitivity, potentially due to variations in acrolein detoxification pathways. However, the mechanistic basis for this differential response remains unresolved.

Urinary concentrations of the stable acrolein metabolite 3-hydroxypropyl mercapturic acid (3-HPMA) exceeded the canine 20 uM threshold in 15 of 74 pet dogs (approximately 20%) in a recent case–control study, with no differences between cases and controls (12). In a separate survey of 41 healthy pet dogs, urinary 3-HPMA levels ranged from 536 to 12,300 ng/mL (equivalent to 2.4–55.6 μM); among these, seven dogs (17%) had concentrations above the genotoxic threshold of 20 uM (10). Although acrolein is unstable, it reaches the urothelium, as evidenced by acrolein-DNA adducts in the bladders of human patients (9). However, the extent to which urinary 3-HPMA concentrations reflect acrolein exposure at the level of the urothelium remains uncertain.

For inorganic arsenic, DNA strand breaks were observed at ≥ 20 uM in canine urinary organoids. This value is comparable to DNA strand breaks seen at 10 uM in a canine immortalized urothelial cell line, a canine primary cell line, and human primary urothelial cells under similar conditions (14). Similarly, 5–10 uM concentrations of sodium arsenite led to DNA strand breaks (24) as well as increased production of reactive oxygen species and oxidized DNA (25) in immortalized human urothelial cells. In contrast to acrolein, DNA strand breaks from arsenite are likely mediated indirectly through reactive oxygen species rather than by direct chemical interactions with DNA (26).

In a recent case–control study of 76 dogs with and without UC, none exhibited total urinary inorganic arsenic concentrations reaching 20 uM (12). Similarly, urinary inorganic arsenic levels in an additional population of 38 healthy pet dogs only ranged from 0.02 to 2.17 uM (10). Only one other study reported canine urinary arsenic concentrations (in untreated shelter dogs; 14.8–18.6 ng/mL); however, inorganic arsenic species were not separately identified and conversion to uM concentrations was not possible from the reported values (27).

For 2,6-DMA, combined strand breaks and oxidative DNA damage were observed in canine urinary bladder organoids at a very low concentration of 0.01 uM. Comparative data in canine or human immortalized or primary urothelial cells were not available. Structural analogs of 2,6-DMA induce mutations in Chinese hamster ovary (CHO) cells at ~ 100 uM concentrations, likely via the generation of reactive oxygen species; however, dose–response data specific to 2,6-DMA were not reported (28).

Among canine UC cases, 8% exhibited urinary 2,6-DMA concentrations at or above 0.01 μM, but none of 36 controls reached this level (13). However, in a separate study of 42 healthy pet and shelter dogs, urinary 2,6-DMA concentrations ranged from 0.05 to 27.2 ng/mL (up to 0.22 μM) (11). Although individual-level data were not available, some of these healthy dogs exceeded the estimated urinary genotoxic threshold for this aromatic amine. Whether urinary 2,6-DMA represents a minor risk factor for canine bladder cancer remains uncertain and would require evaluation in a larger sample size.

This study has important limitations. Testing capacity was limited by the labor-intensive process of generating individual bladder organoids. Only two measures of genotoxicity, DNA strand breaks and oxidized DNA residues, were assessed, and other mechanisms of genotoxicity, including targeted gene methylation and inhibition of DNA repair, should be considered (29). Incubation times of 6 h were based on prior assessments of maximal genotoxic responses over 24 h in canine urothelial cells (14); however, the effects of chronic chemical exposures were not evaluated. Incorporating threshold and sub-threshold concentrations of genotoxic agents over multiple organoid passages might better approximate chronic urothelial exposures and their cumulative impact on DNA integrity (30).

Another limitation of this study is the inability to fully replicate *in vivo* urinary exposures in the *in vitro* model. Urinary acrolein cannot be measured *in vivo* due to its chemical instability, and urinary 3-HPMA might not directly correlate with urothelial acrolein exposures *in vivo*. In addition, it is challenging to assess the genotoxicity of complex mixtures of inorganic arsenic species present *in vivo*, which include arsenite [As(III)], arsenate [As(V)], dimethylarsinic acid (DMA), monomethylarsonic acid (MMA), and trimethylarsine oxide (12). Sodium arsenite [As(III)] was used as a surrogate for total inorganic arsenic in the current *in vitro* assays; however, individual arsenic species differ in their genotoxic potential (31–33).

In summary, the micromolar thresholds for DNA damage from acrolein and inorganic arsenic in canine urinary bladder organoids align with previous findings in immortalized and primary canine urothelial cells. The aromatic amine 2,6-dimethylaniline was genotoxic to canine urinary bladder organoids at nanomolar to micromolar concentrations. Although extrapolating *in vitro* genotoxicity thresholds to *in vivo* urinary exposures presents inherent limitations, some pet dogs are exposed to urinary acrolein or 2,6-DMA concentrations that could lead to early urothelial DNA damage. Whether exposures to the aromatic amine 2,6-DMA contribute to the risk of UC deserves assessment in a larger population of pet dogs. DNA-damaging urinary concentrations of inorganic arsenic appear to be uncommon in pet dogs, based on the samples evaluated to date.

## Data Availability

The original contributions presented in the study are included in the article/supplementary material, further inquiries can be directed to the corresponding author/s.
